# Unlocking Fungal Potential: The CRISPR-Cas System as a Strategy for Secondary Metabolite Discovery

**DOI:** 10.3390/jof10110748

**Published:** 2024-10-29

**Authors:** Karla Leal, Edwind Rojas, David Madariaga, María José Contreras, Kattia Nuñez-Montero, Leticia Barrientos, Olman Goméz-Espinoza, Isabel Iturrieta-González

**Affiliations:** 1 Instituto de Ciencias Aplicadas, Facultad de Ingeniería, Universidad Autónoma de Chile, Temuco 4810101, Chile; karla.leal@uautonoma.cl (K.L.); daavidmt@gmail.com (D.M.); maria.contreras@uautonoma.cl (M.J.C.); 2 Department of Preclinic Sciences, Medicine Faculty, Laboratory of Infectiology and Clinical Immunology, Center of Excellence in Translational Medicine, Scientific and Technological Nucleus (CEMT-BIOREN), Universidad de La Frontera, Temuco 4810296, Chile; e.rojas06@ufromail.cl; 3 Instituto de Ciencias Aplicadas, Facultad de Ciencias de la Salud, Universidad Autónoma de Chile, Temuco 4810101, Chile; kattia.nunez@uautonoma.cl (K.N.-M.); leticia.barrientos@uautonoma.cl (L.B.); 4 Departamento de Ciencias Químicas y Recursos Naturales, Facultad de Ingeniería y Ciencias, Universidad de La Frontera, Temuco 4811230, Chile; olman.gomez@ufrontera.cl; 5 Centro de Investigación en Biotecnología, Escuela de Biología, Instituto Tecnológico de Costa Rica, Cartago 30101, Costa Rica; 6 Jeffrey Modell Center of Diagnosis and Research in Primary Immunodeficiencies, Center of Excellence in Translational Medicine, Medicine Faculty, Universidad de La Frontera, Temuco 4810296, Chile

**Keywords:** fungal natural products, CRISPR-Cas9 genome editing, secondary metabolites synthesis

## Abstract

Natural products (NPs) are crucial for the development of novel antibiotics, anticancer agents, and immunosuppressants. To highlight the ability of fungi to produce structurally diverse NPs, this article focuses on the impact of genome mining and CRISPR-Cas9 technology in uncovering and manipulating the biosynthetic gene clusters (BGCs) responsible for NP synthesis. The CRISPR-Cas9 system, originally identified as a bacterial adaptive immune mechanism, has been adapted for precise genome editing in fungi, enabling targeted modifications, such as gene deletions, insertions, and transcription modulation, without altering the genomic sequence. This review elaborates on various CRISPR-Cas9 systems used in fungi, notably the *Streptococcus pyogenes* type II Cas9 system, and explores advancements in different Cas proteins for fungal genome editing. This review discusses the methodologies employed in CRISPR-Cas9 genome editing of fungi, including guide RNA design, delivery methods, and verification of edited strains. The application of CRISPR-Cas9 has led to enhanced production of secondary metabolites in filamentous fungi, showcasing the potential of this system in biotechnology, medical mycology, and plant pathology. Moreover, this article emphasizes the integration of multi-omics data (genomics, transcriptomics, proteomics, and metabolomics) to validate CRISPR-Cas9 editing effects in fungi. This comprehensive approach aids in understanding molecular changes, identifying off-target effects, and optimizing the editing protocols. Statistical and machine learning techniques are also crucial for analyzing multi-omics data, enabling the development of predictive models and identification of key molecular pathways affected by CRISPR-Cas9 editing. In conclusion, CRISPR-Cas9 technology is a powerful tool for exploring fungal NPs with the potential to accelerate the discovery of novel bioactive compounds. The integration of CRISPR-Cas9 with multi-omics approaches significantly enhances our ability to understand and manipulate fungal genomes for the production of valuable secondary metabolites and for promising new applications in medicine and industry.

## 1. Introduction

Natural products (NPs) significantly contributed to the discovery of pharmacological molecules, such as antibiotics, anticancer, antifungal, antiparasitic, and immunosuppressive compounds [1,2]. Over the past 35 years, the FDA in the United States has approved a range of drugs, more than half of which are based on NPs, distinguished from conventional synthetic molecules by their high structural complexity [3,4]. The unique structural complexity of NPs arises from their in vivo formation, which occurs through multistep enzymatic reactions during the biosynthesis of secondary metabolites in living organisms [5,6]. This biosynthetic form confers NPs advantageous pharmacological properties, such as potential protein interactivity, water solubility, and membrane permeability [7,8]. Therefore, uncovering NPs with novel biological structures and activities is a major challenge in pharmaceutical research.

In this sense, fungi are one of the most significant microbial resources well-suited to address the challenge of discovering new pharmacological molecules [9]. Their significance is attributed to their high capacity to produce structurally diverse and biologically important NPs [10]. Among the fungal-derived NPs discovered are statins (lovastatin), common antibiotics (penicillin, cephalosporin, and amoxicillin), insecticides (nodulisporic acids), and pigments (carotenoids). These compounds have demonstrated the wide applicability of fungal NPs in various medical and industrial sectors [11,12,13]. Notably, the fungal antibiotic amoxicillin is estimated to generate annual worldwide sales exceeding 1 billion dollars [14]. Of the estimated 1.5 million species of fungi on Earth, only about 120,000 have been described, suggesting that approximately 1.38 million species remain unexplored [15]. This vast, largely undescribed diversity has drawn special attention to uncharted habitats, harboring numerous unknown fungi, which are of significant interest in NP biotechnology [16].

Fungal NPs are typically categorized into five groups based on their biosynthetic origin: polyketides, non-ribosomal peptides, terpenoids, alkaloids, and ribosomal peptides or hybrid compounds which incorporate elements from various pathways [17,18,19]. These NPs can vary greatly, usually ranging from C6 to C30 in carbon length, and may include heterocyclic rings and various stereocenters. However, a common feature of these NP pathways is the coordinated and continuous action of several enzymes, each possessing distinct catalytic functions. Among NPs fungal groups, polyketides constitute most fungal NPs and include substances such as statins and a various antibiotic [20,21].

Fungi have managed to preserve numerous functional gene groups over extended periods of time, even though many of them are cryptic or inactive, by employing specific biological and evolutionary processes. These processes have enabled fungi to maintain their cryptic genes, which have been shaped by millions of years of adaptation [22]. One such process is gene duplication, which creates redundant gene copies that can perform new functions without losing their original functions. This process enables the retention of cryptic genes that, although inactive for extended periods, can be reactivated based on environmental conditions, facilitating adaptation to new environments [23]. Additionally, gene duplication promotes functional modularization and specialization, allowing for divergence in regulatory control and specialization in various aspects of fungal biology. This provides a selective advantage of activating cryptic genes in response to specific environmental conditions [24]. For example, a study of the pathogenic fungus *Rhynchosporium commune* revealed that 125 isolates underwent 7879 gene duplications and 116 deletions. Although variations in copy number are often subject to negative selection, duplicated genes were found to be associated with host exploitation, and some duplications contributed to azole resistance and virulence. This suggests that segmental chromosomal duplications are crucial for recent adaptation of this fungus [25]. Furthermore, these genes can be preserved in the genome through mechanisms of purifying selection and conservation strategies, such as “reserve selection”, where cryptic genes act as a genetic reserve to respond to environmental changes [26]. Another evolutionary strategy is the selection of metabolic gene clusters, in which genes related to secondary metabolism are grouped into clusters that can be rapidly activated in response to environmental changes, thereby providing adaptive advantages [27,28]. For example, a study on *Metarhizium anisopliae* in Ontario, Canada revealed that the population structure of this insect-pathogenic fungus is driven by habitat selection rather than host insect selection. Two reproductively isolated groups were identified: one associated with forest habitats, capable of growing in cold conditions (8 °C); and another with agricultural habitats, capable of growing at high temperatures (37 °C) and resistant to UV exposure [29]. An example is the horizontal transfer of a complete sterigmatocystin (ST) gene cluster from *Aspergillus* to *Podospora anserina*. Phylogenetic analysis showed that the genes in the cluster in *Podospora* were highly conserved and aligned with those in *Aspergillus*, despite belonging to different taxonomic classes. Moreover, AflR binding sites were identified, which is a crucial transcription factor, and the expressions of several genes in the cluster were observed across different stages of the *Podospora* lifecycle. This suggests that horizontal transfer resulted in a functional cluster in *Podospora* [30]. The ability of fungi to maintain and activate cryptic genes through evolutionary processes, such as gene duplication, purifying selection, and horizontal gene transfer, showcases their remarkable adaptability to diverse and fluctuating environments. This adaptability makes fungi highly appealing organisms for the production of natural products, as their evolutionary strategies allow them to take advantage of and optimize new genetic functions in response to environmental challenges [31]. This complex biosynthetic diversity in fungi has prompted extensive research into their genetic makeup, leading to groundbreaking discoveries in genome mining.

Over the past few years, genome mining has revealed several gene-encoding enzymes involved in peptide assembly, regulation, resistance, and secondary metabolite synthesis. These genes are organized into sets of genomic structures called “biosynthetic gene clusters” (BGCs), which facilitate the coordinated expression of these pathways [8,32]. The earliest identified BGC in fungi was a 3-gene, 56.9 kb penicillin cluster found in both *Aspergillus nidulans* and *Penicillium rubens* [33,34]. Given their size and the number of proteins required (typically > 40 kb), the formation of NPs is stringently regulated and occurs only when essential for survival. Consequently, most of the putative biosynthetic gene sets of NPs are cryptic and remain silent under laboratory conditions [35]. The increase of genomic knowledge and gene mining from high-throughput sequencing data, coupled with advancements in genomics and transcriptomics, have accelerated the identification and utilization of novel BGCs and secondary metabolites [36]. Therefore, the discovery of new fungal NPs through the prediction of BGCs with bioinformatic tools enables their activation and validation with a multi-omics and synthetic biology approach [37,38].

**Figure 1:**
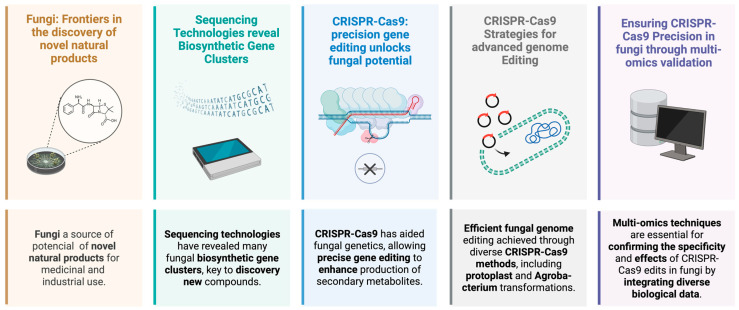
Fungi are the key to discovering pharmacologically active molecules, with advances in genome mining, sequencing, and CRISPR-Cas9 gene editing techniques that reveal and manipulate BGCs for novel compound synthesis. These innovations, along with multi-omics approaches for validation, have significantly enhanced the production and specificity of fungal secondary metabolites, streamlining the pathway to new medical and industrial applications. Created with BioRender.com (accessed on 4 March 2024).

In recent years, advances in new sequencing technologies have unlocked access to more fungal genomes, uncovering new BGCs capable of catalyzing the synthesis of completely novel compounds [39]. Given the cryptic nature of many fungal BGCs, innovative approaches have been employed to activate them [40]. These include heterologous expression and genetic engineering techniques that take advantage of cutting-edge synthetic biology practices. Such methods enable the activation of otherwise dormant BGCs, which are typically unresponsive to environmental stimuli [41]. Within both strategies, genetic engineering for fungal genome editing takes advantage of the host’s native physiology and protein expression system to correctly express BGCs. However, the lack of molecular tools for well-defined regulatory elements in the host poses a challenge for BGC discovery [42] (Figure 1). To address this challenge, researchers have adapted CRISPR/Cas9 (clustered regularly interspaced short palindromic repeats) technology to directly manipulate genomes. Therefore, in the evolving field of mycology, this review compiles insights from studies on the CRISPR-Cas9 system in fungi, aiming to: (i) underscore the significance of CRISPR-Cas systems in advancing fungal genetic research, (ii) provide an overview of the latest advancements in CRISPR-Cas technologies within mycological studies, and (iii) summarize the advances and results of secondary metabolites obtained through this method.

## 2. CRISPR-Cas9 Systems for Genome Editing in Fungi

### 2.1. Types of CRISPR-Cas Systems for Genome Editing in Fungi

There are different types of CRISPR-Cas9 systems that can be used for genome editing in fungi, including the type II Cas9 systems that have been extensively studied in bacteria and eukaryotic cells [43]. The most commonly used CRISPR-Cas9 system for genome editing in fungi is the *Streptococcus pyogenes* type II Cas9 system, which is known for its high specificity and efficiency [44]. Other Cas proteins such as Cas12a and Cas12b are also being explored for their potential use in genome editing in fungi, but these systems are less commonly used at present [45,46]. In addition to genetic modifications, Cas9 can also modulate transcription without modifying the genomic sequence by fusing an enzymatically inactive version of Cas9 (dCas9) with transcriptional activation and repression domains [47,48,49]. It was first used by fusing dCas9 with some repression domains, such as KRAB and Kox1, negatively regulating the transcription of specific genomic loci [50]. This strategy, commonly referred to as CRISPR interference (CRISPRi) decreases transcription of target DNA loci mainly by blocking transcriptional elongation, preventing transcription factor binding, or interfering with the initiation of RNA polymerase transcription [51]. Several studies have shown that this approach can be successfully applied to simultaneously repress transcription of multiple target genes and that it is reversible. For example, Chavez et al. [52] introduced a unique activation strategy that required the fusion of dCas9 with three activation domains, VP64-p65-Rta (VPR) and thus demonstrated its utility in the activation of endogenous coding and non-coding genes. Schüller et al. [53] designed a dCas9-based synthetic RNA-guided transcription activation system for *Aspergillus nidulans* that uses enzymatically inactivated “dead” Cas9 fused to three consecutive activation domains (VPR-dCas9). Furthermore, to avoid negative impacts and facilitate the optimal positioning of VPR-dCas9 RNA at target promoters, they identified nucleosome-free regions. Another variation of gene editing with CRISPR includes prime editing, CRISPEY (CRISPR-enabled prime editing), and base editors (BEs), all of which have shown significant potential in genetic modifications, including in yeast. Prime editing is a genome editing tool based on CRISPR that allows for precise genetic alterations without inducing double-strand breaks (DSBs) [54]. It uses a Cas9 nickase fused to a reverse transcriptase, enabling a range of base pair substitutions, insertions, and deletions with high precision. Prime editing has been successfully applied in various organisms, including yeast, to achieve specific genetic modifications with reduced off-target effects [55]. CRISPEY, another advanced tool, combines the strengths of CRISPR and prime editing to enhance the efficiency and precision of genome editing in yeast. This method has been used to introduce multiple genome alterations and single-nucleotide conversions in yeast, demonstrating its utility in addressing specific genetic challenges and improving various biological systems [56]. Base editors (BEs) are a cutting-edge group of genome editing technologies derived from the CRISPR-Cas system. They enable precise nucleotide modifications without causing DSBs. There are two primary types of base editors: cytosine base editors (CBEs) and adenine base editors (ABEs). CBEs transform cytosine (C) into thymine (T), while ABEs convert adenine (A) to guanine (G) [57]. These tools have demonstrated effectiveness in fungi, allowing for precise genetic modifications with minimal off-target effects [58]. These results demonstrate that this methodology can regulate several genes in parallel and being able to activate individual genes within a set of BGCs that are silent under normal or growth conditions. Thus, dCas9 presents great potential for use in gene expression in filamentous fungi, especially genes associated with the expression and regulation of secondary metabolite biosynthesis [59].

Overall, the choice of CRISPR-Cas9 system for genome editing in fungi would depend on factors such as the fungal species being studied, the desired type of genetic modification, and the efficiency and specificity of the system in that particular context [60]. CRISPR-Cas9 genome editing offers huge potential to accelerate the pace of research in key fungal research areas, such as biotechnology, medical mycology, and plant pathology, by dramatically reducing the time required to undertake common objectives, such as targeted gene deletion, overexpression, single nucleotide changes, or tagging the products of genes of interest with fluorescent proteins [61]. The technique also permits the targeting of gene families, making multiple mutations, and generating mutants in dikaryotic, or polyploid fungi. In the past few years, the CRISPR/Cas9 system has been introduced into filamentous fungi to explore the potential of this strategy in modulating the production of secondary metabolites [61,62]. From *Aspergillus oryzae*, *Trichoderma reesei*, *Aspergillus niger*, and *A. nidulans*, CRISPR/Cas9-based systems have become versatile platforms for precise genome editing, and great progress has already been made toward the production of valuable secondary metabolites [61].

### 2.2. CRISPR-Cas9 Systems

The CRISPR-Cas system is a prokaryotic adaptative immune system that was first described in 1987 when bacteria were found to insert small segments of foreign DNA into their own genomes to recognize and target invading viruses or plasmids [62,63,64]. In 2007, the function of the CRISPR-Cas system as a microbial adaptive immune system was experimentally proven, using the lactic acid bacteria *Streptococcus thermophilus* [65,66,67]. The CRISPR/Cas system functions as an adaptive immune system in numerous *Bacteria* and *Archaea*, of which RNAs harboring previously exposed bacteriophage “spacer” sequences help Cas proteins recognize and cleave specific exogenous DNA. Given that the CRISPR/Cas system presents greater specificity and precision in the selection of sequences, it has become an excellent option for precision genome editing [68,69].

CRISPR/Cas9, a type II CRISPR/Cas system, originally utilizes CRISPR RNA (crRNA) and trans-activating crRNA (tracrRNA) to form a crRNA-tracrRNA duplex [70] This duplex then aids the Cas9 nuclease in recognizing and cutting the target DNA that contains a trinucleotide with a “protospacer adjacent motif” (PAM) and a 5′ end of 20 nucleotides complementary to the spacers. The reprogramming of the system that fuses the crRNA and tracrRNA into a single synthetic guide RNA (sgRNA) significantly enhances the application of the CRISPR/Cas9 system [71]. CRISPR/Cas systems are characterized by a variety of protein components, effector complex structures, genomic location layouts, and mechanisms for adjusting and processing crRNA [72]. CRISPR-Cas systems are generally categorized into two primary groups: Class 1 and Class 2. Class 1 includes effector complexes composed of multiple subunits, whereas Class 2 consists of effector units composed of a single protein, such as Cas9. Within Class 2, Cas12a, formerly known as Cpf1, is an endonuclease belonging to the Type V CRISPR-Cas system [73]. Unlike Cas9, which belongs to the Type II CRISPR systems, Cas12a possesses distinctive features, such as its ability to recognize and cut target DNA sequences by generating staggered cuts, resulting in “sticky ends”. This differs from Cas9, which typically creates blunt ends [74]. In recent times, new subclasses and variants of CRISPR/Cas systems have been discovered, expanding their potential for biotechnological applications, such as genome editing and diagnostic tools that utilize various Cas endonuclease activities [75,76].

Two repair mechanisms, non-homologous end-joining (NHEJ) and homology-directed repair (HDR), have been described to repair DNA DSBs [77]. NHEJ is a repair pathway for DSBs that involves direct ligation of the break ends without using a generally error-prone, RNA-dominant homologous template. For this reason, it can sometimes cause targeted mutations such as random deletions, base substitutions, insertions, targeted chromosomal rearrangements, or frameshift mutations at DNA breakpoints that lead to premature stop codons within the open reading frame of the target gene [71]. In contrast, HDR is a less efficient but high-fidelity and less common pathway in microorganisms than NHEJ [78]. HDR accurately repairs DSBs via a homologous DNA template or an exogenous donor fragment and thus has the potential to generate genetic modifications through desired gene insertions or induction of nucleotide substitutions [79]. Since then, the reprogrammed CRISPR/Cas9 system has been successfully used in the editing of *Saccharomyces cerevisiae*, *Drosophila melanogaster*, *Caenorhabditis elegans*, and plant and human embryos [47,80].

CRISPR/Cas9 has revolutionized genetic editing in fungi due to its remarkable efficiency, versatility, and ease of use. This system is favored over traditional gene-editing methods such as homologous recombination, zinc finger nucleases, and transcription activator-like effector nucleases because of its high targeting efficiency, straightforward primer design, and broad applicability. These advantages have made CRISPR/Cas9 the leading genomic editing technology in both academic research and industrial applications [81] It enables precise and simultaneous editing of multiple genes, aiding the genetic manipulation of thermophilic fungi like *Myceliophthora thermophila* and *M. heterothallica*, thereby significantly increasing cellulase production, this method requires only the Cas9 protein and a sgRNA, simplifying its use compared to other genetic editing methods [82]. In filamentous fungi such as *Pyricularia oryzae*, it efficiently recognizes and edits endogenous genes [83]. CRISPR/Cas9 has accelerated industrial applications by developing improved strains for enzymes, biofuels, and chemical production. In *Aspergillus niger*, it has been used to mutate genes, enhancing plant biomass degradation [84]. Additionally, it has advanced genetic research by facilitating gene manipulation to study functions and improve traits. In the pathogenic fungus *Candida albicans*, CRISPR/Cas9 has created specific mutations, aiding new therapy development [85].

Regarding the efficacy of CRISPR gene editing, this technology can lead to unwanted mutations in genomic regions that share similarities with the target site, which can negatively impact experimental results [86]. Additionally, sgRNA can bind to DNA sequences that are not fully complementary, resulting in off-target cuts by the Cas9 enzyme [87]. Chemical modification of sgRNAs has markedly enhanced genome editing efficiency, particularly in human primary cells, by boosting process efficacy and minimizing toxicity linked to DNA delivery methods [88]. Despite this, accurately predicting sgRNA efficacy remains complex due to the modest correlation between predicted and actual activities. Key factors like GC content at the PAM distal end have been identified, indicating that sgRNA stability and efficacy can be improved through meticulous sequential design and advanced prediction algorithms [89]. The efficiency of RNP complexes, including sgRNAs, is also highly dependent on the delivery method. Innovations in delivery methods, such as nanoparticles and chemical carriers, have shown significant promise in enhancing the stability and efficacy of sgRNA-Cas9 complexes, improving gene editing outcomes [90]. However, in fungi deficient in NHEJ repair, a decrease in these off-target mutations has been observed, suggesting that this repair mechanism contributes to the occurrence of these unwanted effects [91]. Despite these challenges, the CRISPR/Cas9 system has enabled precise genetic modifications in fungi, facilitating genetic research and the improvement of industrial strains through the insertion, deletion, or modification of specific genes [83,92]. The Cas9 enzyme can efficiently create double-strand breaks in DNA, allowing the integration of sequences of interest through homologous recombination [93]. Additionally, it permits the simultaneous editing of multiple genes, which is beneficial for functional genetics studies and the development of strains with multiple phenotypic improvements [94]. The utilization of modified Cas9 variants, such as SpCas9-HF1, eSpCas9, HypaCas9, Cas9-NG, and xCas9, can diminish off-target effects in genetic modifications employing CRISPR [95]. Recently, the SpRY variant has been developed, which virtually eradicates the necessity for a PAM for SpCas9 in human cells, while significantly lessening detectable off-target effects [96]. These variants not only reduce off-target effects but also expand the range of compatible PAM sequences, surpassing the original limitation of the PAM requirement for DNA-targeted CRISPR enzymes. In this context, PAM-less Cas9 proteins, such as SpRY, provide considerable enhancements in gene editing due to their greater targeting flexibility and improved accessibility to target sites [97]. By not requiring a PAM, they can target a broader range of sites in the genome, allowing for more versatility in gene editing applications. In addition, these proteins enhance the modification of genetic regions that were previously challenging to address with conventional Cas9 proteins, broadening their usefulness in both research and therapeutic settings. PAM-less variants have been designed to improve specificity, reducing the likelihood of unintended cuts, and increasing the accuracy of genetic modifications [96,98]. However, PAM-less Cas9 variants also have certain limitations that should be considered when using them. Firstly, they exhibit reduced activity in specific regions of the genome, which can compromise the efficiency of gene editing and necessitate specific optimization [99]. Furthermore, the development and optimization of these variants involve complex processes, such as extensive modifications and testing, which can be costly and time-consuming. Moreover, the effectiveness of these variants can vary considerably between different organisms and genomic contexts, potentially displaying less robust performance in fungal genomes compared to human cells, necessitating additional adjustments and optimization [100,101].

In fungi, SpCas9-NG has been used in *S. cerevisiae* for multiple genome alterations and single-nucleotide conversions, demonstrating the ability of Cas9 variants to address specific challenges and improve the accuracy of various biological systems [56].

### 2.3. Methodologies in CRISPR-Cas9 Genome Editing of Fungi

Genetic engineering of filamentous fungi using the CRISPR-Cas9 technique has experienced remarkable progress over the years. There are multiple alternatives and variations in protocols, which are adjusted according to the specific type of fungus and research objectives [62]. However, there are a number of general steps and considerations that are common to these procedures, such as designing the sgRNA, delivery and gene editing by CRISPR system, and verification of edited strains. The sgRNA design is a critical step in the CRISPR-Cas9 methodology for genome editing in filamentous fungi. It involves the identification of a target sequence within the fungal genome that precedes a PAM and the synthesis of a complementary RNA sequence that will guide the Cas9 enzyme to the desired genomic location [71]. The efficiency and specificity of the CRISPR-Cas9 system rely heavily on the gRNA’s ability to match the target DNA sequence without off-target effects. Several bioinformatics tools, such as CRISPOR, sgRNAcas9, and E-CRISP, have been developed to predict sgRNA specificity and potential off-target sites [102,103]. Optional design validation can be performed using in vitro assays or deep sequencing to ensure the accuracy of the sgRNA design before fungal transformation [104]. The Cas9 gene and sgRNAs are then cloned into vectors suitable for their purpose. These genetic constructs are introduced into fungal cells using specific transformation techniques, such as protoplast transformation, electroporation, or *Agrobacterium*-mediated transformation, depending on the particularities of the target fungus and the research objectives. The protoplast-polyethylene glycol (PEG)-mediated transformation method is a fundamental approach for introducing the CRISPR-Cas system into filamentous fungi [105]. This process begins with the enzymatic breakdown of fungal cell walls to create protoplasts, which are subsequently treated with PEG to enhance the incorporation of the CRISPR-Cas9 DNA construct into fungal cells, thereby facilitating precise genomic alterations [106]. Using this approach, the research aimed to investigate the potential application of the CRISPR-Cas9 system in *Nodulisporium* sp., as well as other strains like *Aspergillus oryzae* NSAR1 and *Sporormiella minima* (No. 40-1-4-1). The study developed an effective gene disruption strategy, enhancing efficiency by inserting a genetic cassette at the Cas9 cleavage site. Through CRISPR editing in *Nodulisporium* sp., new metabolites were induced, mainly compounds related to demetoxicovidin and its derivatives, which demonstrated significant activity against Alzheimer’s disease [107]. Electroporation offers an alternative strategy by harnessing electrical pulses to transiently permeabilize fungal cell membranes, thereby allowing the direct introduction of RNP complexes consisting of Cas9 protein and guiding RNA into fungal cells [108]. This method avoids the need for stable DNA integration and improves gene editing precision. Another methodology used is Agrobacterium-mediated transformation (ATMT), which is a widely adopted method for recruiting the CRISPR-Cas9 system in filamentous fungi. This technique is based on the use of *Agrobacterium tumefaciens* to transfer a T-DNA vector containing the CRISPR-Cas9 construct into fungal cells, thereby exploiting the natural genetic exchange mechanisms of bacteria [109]. Moon et al. [110] is a significant scientific example illustrating the application of *Agrobacterium*-mediated transformation in fungi for genomic manipulation. Their work entailed selective disruption of a gene implicated in the biosynthesis of non-ribosomal peptides within *Metarhizium anisopliae*. This disruption was achieved through targeted genetic engineering, a methodology with broad implications for modifying fungal genomes to study gene function and regulation.

Evaluation of the verification of edited strains by CRISPR-Cas9 in filamentous fungi is a critical component of genome editing, leveraging both sequencing and various biological techniques to confirm and characterize the edits. Sequencing methods, such as Sanger sequencing and next-generation sequencing (NGS), are pivotal for verifying precise genetic modifications at the target site, ensuring the specificity and efficacy of CRISPR editing [111,112]. Additionally, biological assays, including phenotypic analysis, quantitative PCR (qPCR), and Southern blotting, need to be employed to assess the functional impact of the mutations and detect any off-target effects [113]. For instance, in *Aspergillus nidulans*, CRISPR-Cas9 was used to activate silent BGCs, and sequencing confirmed the edits and subsequent metabolite analysis revealed new secondary [114].

## 3. Advances in the Application of CRISPR-Cas9 for the Production of Secondary Metabolites in Fungi

In recent years, CRISPR/Cas9-based systems have been used as versatile editing and modulation platforms for fungal genomes. In addition, CRISPR/Cas9 technologies have been specifically adapted for filamentous fungi, unlocking their potential for enhanced activation and the production of secondary metabolites (Table 1). Therefore, the CRISPR/Cas9 system is currently recognized as a powerful tool for gene editing that has been studied in diverse species of fungi, achieving gene inactivation in more than 40 species of filamentous fungi and oomycetes, and providing an opportunity to characterize gene functions related to the virulence, pathogenicity, growth, and reproduction in fungus [115]. CRISPR systems for gene activation, as well as CRISPR/Cas9 systems used for gene insertions, substitutions, and deletions, are the most commonly used methods in filamentous fungi [116,117].

### 3.1. CRISPR-Cas9 Gene Editing System

CRISPR/Cas9 cutting systems allow the expression of genes responsible for fungal metabolite production. Engineered Cas nucleases have expanded the set of tools available for gene editing. Cas9 features two independent nuclease domains, which can be modified to abolish DNA cutting ability while retaining RNA-guided DNA binding ability [118]. In contrast, Cas9 nickase was developed by inactivating a single nuclease domain, generating a mutant capable of cutting only one DNA strand. In addition to nickases, fully nuclease-inactivated variants (dCas) have been identified. Cas9 nuclease and dCas proteins have served as the basis for base-editing technologies, where they can be fused with a deaminase enzyme responsible for nucleotide conversions [119]. In filamentous fungi such as *A. niger* and *Myceliophthora thermophila*, dCas9 cytosine base editors capable of transforming C into T have been used [42,120].

In the last few years, several papers have been published, such as Chen et al. [121] demonstrating the successful expression of the optimized Cas9 codon, which was used together with the recently discovered promoter PCMLSM3 and terminator TCMURA3. Furthermore, by implementing the negative selection marker URA3, they developed a CRISPR-Cas9 system that incorporates Cas9 DNA endonuclease, in vitro pre-synthesized RNA, and a single-stranded DNA template to efficiently generate site-specific deletions and insertions. In addition, this method was found to be faster and more efficient than previous methods used to edit fungal genes, showing that the CRISPR-Cas9 system is a valuable tool for gene editing in *Cordyceps militaris*, which has important implications for the production of food and drugs derived from this fungus. The authors suggested that this technology could be used to further improve the nutritional and medicinal properties of *C. militaris* [122]. Other work reported by Dong et al. [82] enhanced the expression of thermostable trehalase from *Myceliophthora sepedonium* in *A. niger*. Trehalase is an enzyme that catalyzes the conversion of one molecule of trehalose into two molecules of glucose. In this study, the authors used the CRISPR/Cas9 tool to achieve successful overexpression of TreM trehalase by a multiple insertion strategy. The result was a recombinant strain named Du-TreM that exhibited the highest trehalase activity (4329.58 U/mL) after 168 h of fermentation and medium optimization, 4.8-fold than the transformant obtained via the traditional method. In addition, the authors performed purification and characterization of recombinant TreM by gel filtration chromatography and obtained satisfactory results. They found that TreM had a temperature optimum at 70 °C and a pH optimum at 5.5. This study demonstrates that the use of CRISPR/Cas9 can significantly improve heterologous protein expression in *A. niger* and that recombinant TreM has thermostable and acidic properties useful for industrial applications. In this regard, a paper by Jimenez et al. [123] performed on the industrial mushroom *Ashbya gossypii* used the CRISPR/Cas9 system with a single vector. The main objective of this study was to expand the set of molecular tools available for genomic manipulation of this fungus, which is used in the industrial production of vitamin B2 and other high value-added compounds. The researchers designed and constructed an expression vector containing both the CRISPR/Cas9 system and a guide sequence specific to the target gene. This vector was introduced into *A. gossypii* cells to allow precise genome editing. The results showed that this single-vector system achieved high efficiency in gene inactivation and the introduction of point mutations into the *A. gossypii* genome. In addition, this system was shown to be capable of generating mutants with the desired phenotypes, demonstrating its utility in the genetic engineering of this industrial fungus [124].

Along with this, more current works such as the one carried out by Chen et al. [125] developed an efficient and robust genetic manipulation system in two marine fungi, *Spiromastix* sp. SCSIO F190, and *Aspergillus* sp. SCSIO SX7S7. The focus was on the use of the CRISPR-Cas9 system to achieve targeted gene disruption in these fungi and trigger the production of novel secondary metabolites. First, a PEG-mediated chemical transformation system was established for the protoplasts of the two aforementioned marine fungi. A CRISPR-Cas9-based gene disruption strategy was then developed by transforming the target fungi with a single circular plasmid. This plasmid encoded Cas9, a sgRNA, and selectable marker. This transformation resulted in a high frequency of targeted and insertional gene mutations in both the marine fungi. One of the genes mutated using the established CRISPR-Cas9 system was the histone deacetylase gene RPD3. This mutation activated the production of novel secondary metabolites that were not produced by the wild-type fungal strain. These secondary metabolites have potential applications in a variety of fields, so these are promising results for industrial applications [126]. The research into the use of the microhomology-mediated end joining (MMEJ) technique in *Aspergillus fumigatus* has demonstrated successful integration of an exogenous GFP tag and precise editing of genes such as pksP and cnaA. This editing made it possible to investigate the effects of mutations on the production of secondary metabolites. One of these secondary metabolites is *Conidial Melanin*, whose production is impacted by the mutation in the pksP gene, while the mutation in the cnaA gene affects the function of the catalytic subunit of calcineurin, which can influence the biosynthesis of various secondary metabolites involved in the fungus’s adaptation and virulence [127]. Similarly, an efficient CRISPR-Cas9 system was established for gene inactivation in *Alternaria alternata*. The pksA and brm2 genes from the melanin biosynthesis pathway were selected as targets, resulting in several white mutants after multiple rounds of strain purification. Additionally, an uracil auxotrophic strain was created by inactivating the pyrG gene. The mutation of the brm2 gene led to a decrease in melanin production, thereby affecting the biosynthesis of this important secondary metabolite. These results demonstrate the potential of CRISPR/Cas9 technology for precise genetic manipulation in *A. alternata*, opening new avenues for studying and exploiting fungal secondary metabolism [128].

### 3.2. CRISPR Activation/Repressor System

Based on the origin and regulation of the BGCs of interest, some researchers have used dCas9-based activation or repression systems because of their versatility in targeting one or more gene locations easily and simultaneously by simply expressing an appropriate combination of guide RNAs [129] CRISPR transcriptional and epigenetic regulators have been used to manipulate BGCs in certain *Aspergillus* and *Penicillium* species. These tools enable transcriptional modification without requiring programmed gene editing. One study focused on the use of CRISPR/dCas9 technology to study epigenetic modifications in secondary metabolites produced by *A. niger*. The authors demonstrated that the p300-dCas9 protein fusion can specifically increase the expression of secondary metabolic genes at specific sites, which in turn increases the production of fumonisin B2. In addition, the authors also demonstrated that endogenous GcnE-dCas9, RpdA-dCas9, and HosA-dCas9 proteins can effectively repress gene transcription [130]. Overall, these results suggest that CRISPR/dCas9 is a useful tool for studying epigenetic modifications in *A. niger* and may have important implications for the development of new drugs or biotechnological applications.

Another methodology used is the CRISPRa. Roux et al. [131] used this technique to activate biosynthetic gene clusters in two different species of filamentous fungi and analyzed the metabolites produced by these modified strains. The methodology used in this study included the construction of a CRISPR/dLbCas12a-VPR system for the CRISPR-mediated activation of biosynthetic gene clusters in *A. nidulans* and *P. chrysogenum*. In addition, they used techniques such as quantitative real-time PCR and liquid chromatography-mass spectrometry to analyze gene expression and metabolite production in modified strains. The results showed that CRISPR-mediated activation of BGCs in filamentous fungi could be a useful tool for discovering new bioactive molecules. Several new and known metabolites produced by modified strains have been identified, including sorbicillinoids, which are yellow pigments with significant bioactivity, including antiviral, anti-inflammatory, and antimicrobial properties. Researchers have identified a crucial gene, Pc21g05080 (pks13), which encodes a polyketide synthase (PKS) essential for the biosynthesis of sorbicillinoids. This PKS is responsible for the production of polyketide precursors, such as sorbicillin and dihydrosorbicillin, which are subsequently processed into various derivatives, including bisorbicillinoids. These results suggest that this technique can be used to discover new compounds with therapeutic and agricultural potential.

### 3.3. CRISPR-Cas9 Ribonucleoproteins

In certain fungal species or strains, efficient expression of Cas endonuclease cassettes and sgRNA may be limited. To overcome this challenge, an alternative method has been identified that involves the introduction of the Cas/sgRNA complex into the fungal nucleus by in vitro transformation of pre-assembled RNP [132]. The CRISPR system using RNP stands out compared to DNA-based CRISPR systems, as the RNP approach eliminates the need for strain-specific construction and can be applied in a versatile manner across diverse species and strains [133,134]. The first successful use of RNP delivery in filamentous fungi was demonstrated in *Aspergillus fumigatus*, where microhomology editing of just 30 base pairs was achieved, showing significantly greater gene-targeting efficiency in various genetic contexts of *A. fumigatus* than traditional gene replacement systems [95]. An efficient gene editing system was created using improved Cas9 RNP complexes to modify the genes of *Fusarium oxysporum*. The protoplast transformation process facilitated by PEG enabled the mutation of genes like URA5 and URA3, resulting in the development of uracil auxotrophic mutants and resistance to 5-fluoroorotic acid (5-FOA). Additionally, the BIK1 gene was altered, demonstrating its role in the synthesis of bikaverin, a red secondary metabolite, with an efficiency of up to 50% [135]. Another example is in *Epichloë* species, where the modified Cas9 nuclease RNP complex and single guide RNA pairs were used to eliminate the entire ergot alkaloid biosynthesis cluster, which prevented the production of secondary metabolites toxic to livestock [136]. Since then, this approach has been employed to edit BGCs in various fungal species, such as *Penicillium decumbens*, *Fusarium proliferatum*, *Penicillium polonicum*, *Metarhizium brunneum*, *Mucor circinelloides*, and *Epichloë coenophiala* [137,138,139,140]. In a study by David et al. the CRISPR-Cas9 technique was used together with heterologous expression to optimize the production of the ergot alkaloids lysergic acid (LA) and dihydrolysergic acid (DHLA) in the fungus *Metarhizium brunneum*. Ergot alkaloids are critical specialized metabolites used in neuropharmaceuticals. This alternative organism outperformed *Neosartorya fumigata* in terms of performance, achieving a notable improvement in the secretion of these compounds into the culture medium, which was significantly more efficient than previous attempts with *N. fumigate* [138].

It is important to underline that RNPs are present transiently, thus reducing the risk of off-target effects. Since no exogenous DNA is required for CRISPR/Cas expression, this strategy is favorably considered for editing without the introduction of transgenes. However, the efficacy of RNP editing may be limited by the instability and susceptibility of sgRNA during fungal transformation. Alternatively, co-transformation with marker amplicons or marker-bearing plasmids can be used to identify the transformation events. Marker-free editing has also been achieved through RNP delivery [141]. As well, another approach developed by Pohl et al. 2016 was based on in vivo recombination of an AMA1 plasmid to restore a marker, which could subsequently be removed by culturing on nonselective media [142].

**Table 1:** CRISPR-cas9 gene editing technology in filamentous fungi to explore its potential for activation and secondary metabolite production.

Author	Fungus Species	CRISPR Methodology	Outcome		Metabolites Altered	Industrial Importance	Breakthrough Technologies
Abdallah et al. [143]	*Aspergillus* *fumigatus*	CRISPR-Cas9 RNPs	Greater gene-targeting efficiency across different genetic backgrounds; improved efficiency in multiple clinical isolates		General metabolism	Potential implications in medical mycology	Use of RNPs for enhanced gene editing
Cao et al. [144]	*Scheffersomces* *stipitis*	CRISPR-Cas9	Improved HDR-based genome editing efficiency		Increased production of shikimate pathway derivatives	Relevance in biofuel production	Optimization of HDR efficiency
Chen et al. [121]	*Cordyceps* *militaris*	CRISPR-Cas9	Efficient site-specific deletions and insertions, enhancing gene editing		Increased production of lactic acid	Application in pharmaceuticals and food	Use of synthetic RNA and single-stranded DNA templates
Chen et al. [125]	*Spiromastix* sp., *Aspergillus* sp.	CRISPR-Cas9	Activation of novel secondary metabolites in marine fungi through targeted gene disruption		Increased production of novel, unidentified secondary metabolites	Potential applications in medicine	CRISPR-based gene disruption strategy
Deng et al. [145]	*Shiraia* *bambusicola*	CRISPR-Cas9	Reduction of sorbicillinoid levels and increased cephalosporin C production		Reduced sorbicillinoid, increased cephalosporin C	Relevance in antibiotic production	CRISPR-based targeted gene disruption
Dong et al. [120]	*Aspergillus* *niger*	CRISPR-Cas9	Successful overexpression of thermostable trehalase, improving protein expression		Ethanol increase	Industrial applications in enzyme production	Multiple insertion strategy
Florea et al. [136]	*Epichloë species*	CRISPR-Cas9 RNP	Elimination of entire ergot alkaloid biosynthesis cluster, preventing the production of toxins		Elimination of ergot alkaloids	Importance in agriculture (livestock safety)	Use of RNPs for gene cluster elimination
Hicks et al. [146]	*Fusarium* *graminearum*	CRISPR-Cas9	Disruption of PKS and terpene synthase genes, leading to the production of new metabolites		Increased decalin-containing diterpenoid pyrones, FDDP-D and FDDP-E.	Implications in agriculture and medicine	Targeted gene disruption for metabolite profiling
Li et al. [130]	*Aspergillus* *niger*	dCas9 cytosine base editors	Transformation of cytidine into thymine using dCas9 cytosine base editors		The expression of fwnA and accelerated the synthesis of melanin.	Potential in genetics research	Base editing technology for precise nucleotide conversions
Jiménez et al. [123]	*Ashbya gossypii*	CRISPR-Cas9	High-efficiency gene inactivation and point mutations, aiding in the genetic engineering		General metabolism	Relevance in vitamin B2 production	Single-vector system for CRISPR delivery
Katayama et al. [100]	*A. oryzae*, *A. sojae*	CRISPR-Cas12a	Marker-free mutagenesis of AowA, sC genes in A. oryzae, and AswA gene in A. sojae		General metabolism	Applications in Food industry	Marker-free CRISPR-Cas12a mutagenesis
Leisen et al. [134]	*Botrytis cinerea*	CRISPR-associated marker-free editing	Marker-free gene editing for biosynthesis of phytotoxic compounds		New amino acid substitutions that conferred varying levels of resistance to different SDHI fungicides.	Agricultural applications (plant pathology)	Development of marker-free gene editing tools
Li et al. [130]	*Aspergillus* *niger*	CRISPR/dCas9	Activation of breF and fwnA; Repression of breF; Increased fumonisin B2 production; Accelerated melanin synthesis		Increased production of the compound fumonisin B2, accelerated the synthesis of melanin	Relevance in mycotoxin control	Use of dCas9 for transcriptional activation/repression
Pohl et al. [142]	*Penicillium* *rubens*	CRISPR-Cas9	Creation of a SM-deficient strain as a platform for heterologous gene cluster integration, resulting in the production of decumbenones		Increased decumbenone A, B, and C	Industrial production of novel metabolites	Heterologous gene cluster integration
Wang et al. [135]		*Fusarium* *oxysporum*	CRISPR-Cas9 RNP	Generated uracil auxotrophic mutants and resistance to 5-fluoroorotic acid; Mutation of BIK1 gene confirmed role in bikaverin synthesis	Increased Bikaverin	Potential in pigment and antibiotic production	Use of RNPs for efficient gene targeting
Wenderoth et al. [128]		*Alternaria* *alternata*	CRISPR-Cas9	Created uracil auxotrophic strain by inactivating pyrG gene; Mutation of brm2 gene decreased melanin production	Decreased melanin production	Relevance in the study of fungal pathogenicity	Efficient CRISPR-based gene inactivation

RNP: Ribonucleoprotein; HDR: Homology-Directed Repair; PKS: Polyketide Synthase; SDHI: Succinate Dehydrogenase Inhibitor; dCas9: Dead Cas9; CRISPR: Clustered Regularly Interspaced Short Palindromic Repeats; Cas: CRISPR-associated protein; SM: Secondary Metabolite.

## 4. Integration of Multi-Omics Knowledge for the Validation of CRISPR-Cas9 Technique

The integration of multi-omics knowledge can enhance the validation of the CRISPR-Cas9 technique in fungi by providing a comprehensive understanding of its effects at genomic, transcriptomic, proteomic, and metabolomic levels. This integration allows for the identification of target sites, assessment of functional consequences, identification of off-target effects, and optimization of editing protocols. In addition, statistical and machine learning approaches play a crucial role in analyzing and interpreting multi-omics data, enabling researchers to gain valuable insights from integrated fungal datasets [60].

Genomic data is important to identify suitable target sites for CRISPR-Cas9 editing. By analyzing the genome sequence of the fungus, researchers can identify regions that are highly specific and have low off-target effects [147]. Moreover, the integration of genomic data with other omics data types can help prioritize target sites based on their functional relevance in fungi. Xie et al. [148] by genomic data mining of *Poria cocos*, identified the endogenous U6 promoters and the promoter sequence was used to construct a sgRNA expression vector pFC332-PcU6. Next, the protoplast isolation protocol was developed, and the sgRNA-Cas9 vector was successfully transformed into *P. cocos* cells by a PEG/CaCl_2_-mediated transformation method. Non-target sites were predicted and detected genome-wide. As a result, the URA3 target marker gene was successfully disrupted by the CRISPR-Cas9 system [148]. The first report of genomic editing in *P. cocos* using the CRISPR/Cas9 system that integrates genome-wide prediction and detection of off-target sites.

On the other hand, transcriptomic data can provide valuable information on the effects of CRISPR-Cas9 editing at the fungal transcript level. By comparing the gene expression profiles before and after CRISPR-Cas9 editing, researchers can identify differentially expressed genes and understand the functional consequences of targeted genetic modifications [149]. Integration of transcriptomic data with genomic and proteomic data can validate the effectiveness of CRISPR/Cas9 editing and provide a comprehensive understanding of the molecular changes induced by this technique in fungi [150]. Run et al. [151] conducted an efficient targeted point sequence based on the gene transcriptome data of *Flammulina velutipes* mycelium and primordium stages. Proteomic and metabolomic data could further elucidate the downstream effects of CRISPR-Cas9 editing in fungi. By analyzing changes in protein expression and metabolite levels, researchers can gain insights into the functional consequences of targeted genetic modifications at the protein and metabolic pathway levels [78]. A study carried out by Hicks et al. [146] using CRISPR-Cas9 gene-editing experiments disrupted the PKS and terpene synthase genes associated with the C16 biosynthetic gene cluster in *F. graminearum*. Culture media screening experiments using transformant strains were profiled by UHPLC-HRMS and targeted MS2 experiments to confirm secondary metabolite products associated with the C16 biosynthetic gene cluster, such as decalin-containing diterpenoid pyrones, FDDP-D, and FDDP-E. The production of both decalin-containing diterpenoid pyrones was confirmed in wheat heads attacked by *F. graminearum* using growth chamber assays. Integration of proteomic and metabolomic data with genomic and transcriptomic data can provide a comprehensive view of the molecular changes induced by CRISPR/Cas9 editing and help validate the effectiveness of this technique in fungi. This is particularly useful for BGCs editing where genetic changes might have effects on metabolic pathways and production of related secondary metabolites. Multi-omics approaches are also necessary to evaluate the off-target effects observed with the use of Cas nucleases [152]. By comparing the genomic, transcriptomic, proteomic, and metabolomic profiles of edited fungal organisms with those of control samples, researchers can identify unintended changes induced by CRISPR-Cas9 [153]. This integration can help assess the specificity and potential side effects of the technique and guide the optimization of target site selection and editing protocols for fungi.

Finally, statistical and machine learning (ML) approaches can be effectively integrated to analyze multi-omics data on fungi [154]. The most commonly employed ML algorithms in biological data analysis include artificial neural networks (ANN) [155], Naive Bayes [21], support vector machines (SVM) [156] C4.5 decision trees [157], k-nearest neighbors (KNN) [158], and regression [159]. Two different strategies have been employed in silico. The first strategy focuses on identifying BGCs, where the data-mining process locates genes with conserved enzymes linked to secondary metabolism. The second strategy is used to confirm the identity of the natural products in various classes [160]. These methods can help identify patterns, correlations, and regulatory networks within the data, enabling the identification of key molecular players and signaling pathways affected by CRISPR-Cas9 editing. Integrative approaches can also facilitate the development of predictive models for assessing the efficacy and safety of CRISPR-Cas9 editing in fungi.

## 5. Conclusions

The exploration of fungal NPs for pharmacological applications has gained prominence owing to their structural complexity and advantageous pharmacological properties. Fungi, with their vast, largely unexplored diversity, are a rich source of NPs, including antibiotics, statins, and insecticides. The biosynthetic pathways for these compounds are encoded by BGCs, and advancements in genome mining have accelerated the identification of novel BGCs. The discovery and activation of these BGCs have been revolutionized by the application of CRISPR-Cas9 systems in fungi. CRISPR-Cas9, originally a prokaryotic immune system, has been used for precise genome editing in fungi. Different Cas9 variants, including the widely used *Streptococcus pyogenes* Cas9, offer versatile options for editing fungal genomes. The applications of the system extend beyond traditional gene editing, as demonstrated by CRISPRi and CRISPRa techniques, allowing for targeted gene repression or activation. Moreover, the delivery of CRISPR components such as RNPs has emerged as an efficient strategy to minimize off-target effects and expand the range of compatible PAM sequences. The integration of multi-omics data, encompassing genomics, transcriptomics, proteomics, and metabolomics, has enhanced the validation of CRISPR-Cas9 techniques. This integrated approach enables a comprehensive understanding of the molecular changes induced by CRISPR-Cas9, aids in identifying off-target effects, and guides the optimization of editing protocols. Statistical and machine-learning approaches further contribute to data analysis, pattern recognition, and the development of predictive models.

In recent years, CRISPR/Cas9 has been successfully applied to various filamentous fungi, including *Aspergillus*, *Trichoderma*, and *Fusarium* species, demonstrating its potential for accelerating research in biotechnology, medical mycology, and plant pathology. The ability of this technique to modulate secondary metabolite production in fungi holds promise for the discovery of novel bioactive compounds with applications in medicine and agriculture. Ongoing advancements in CRISPR-Cas9 technology and its integration with multi-omics approaches have made it a powerful tool for unlocking the vast potential of fungal natural products.

## Abbreviations

NPs	Natural Products
FDA	Food and Drug Administration
BGCs	Biosynthetic Gene Clusters
CRISPR	Clustered Regularly Interspaced Short Palindromic Repeats
Cas9	CRISPR-Associated Protein 9
sgRNA	Single Guide RNA
dCas9	Dead Cas9 (catalytically inactive version of Cas9)
HDR	Homology-Directed Repair
NHEJ	Non-Homologous End Joining
PAM	Protospacer Adjacent Motif
CRISPRi	CRISPR Interference
CRISPRa	CRISPR Activation
RNP	Ribonucleoprotein
PEG	Polyethylene Glycol
PKS	Polyketide Synthase
GFP	Green Fluorescent Protein
HR	Homologous Recombination
5-FOA	5-Fluoroorotic Acid
MMEJ	Microhomology-Mediated End Joining
CRISPEY	CRISPR-Enabled Prime Editing
CBEs	Cytosine Base Editors
ABEs	Adenine Base Editors
NGS	Next-Generation Sequencing
qPCR	Quantitative Polymerase Chain Reaction
ANN	Artificial Neural Network
SVM	Support Vector Machine
KNN	K-Nearest Neighbors
